# The Role of the N-Terminal Domains of Bacterial Initiator DnaA in the Assembly and Regulation of the Bacterial Replication Initiation Complex

**DOI:** 10.3390/genes8050136

**Published:** 2017-05-10

**Authors:** Anna Zawilak-Pawlik, Małgorzata Nowaczyk, Jolanta Zakrzewska-Czerwińska

**Affiliations:** 1 Hirszfeld Institute of Immunology and Experimental Therapy, Polish Academy of Sciences, Weigla 12, Wroclaw 53-114, Poland; malgorzata.nowaczyk@iitd.pan.wroc.pl (M.N.); jolanta.zakrzewska@uni.wroc.pl (J.Z.-C.); 2 Department of Molecular Microbiology, Faculty of Biotechnology, University of Wrocław, ul. Joliot-Curie 14A, Wrocław 50-383, Poland

**Keywords:** DnaA, N-terminus of DnaA, *oriC*, chromosomal replication, orisome, HobA, DiaA, SirA, Hda, Dps, DnaB

## Abstract

The primary role of the bacterial protein DnaA is to initiate chromosomal replication. The DnaA protein binds to DNA at the origin of chromosomal replication (*oriC*) and assembles into a filament that unwinds double-stranded DNA. Through interaction with various other proteins, DnaA also controls the frequency and/or timing of chromosomal replication at the initiation step. *Escherichia coli* DnaA also recruits DnaB helicase, which is present in unwound single-stranded DNA and in turn recruits other protein machinery for replication. Additionally, DnaA regulates the expression of certain genes in *E. coli* and a few other species. Acting as a multifunctional factor, DnaA is composed of four domains that have distinct, mutually dependent roles. For example, C-terminal domain IV interacts with double-stranded DnaA boxes. Domain III drives ATP-dependent oligomerization, allowing the protein to form a filament that unwinds DNA and subsequently binds to and stabilizes single-stranded DNA in the initial replication bubble; this domain also interacts with multiple proteins that control oligomerization. Domain II constitutes a flexible linker between C-terminal domains III–IV and N-terminal domain I, which mediates intermolecular interactions between DnaA and binds to other proteins that affect DnaA activity and/or formation of the initiation complex. Of these four domains, the role of the N-terminus (domains I–II) in the assembly of the initiation complex is the least understood and appears to be the most species-dependent region of the protein. Thus, in this review, we focus on the function of the N-terminus of DnaA in orisome formation and the regulation of its activity in the initiation complex in different bacteria.

## 1. Introduction

Chromosomal replication is a key step in cell cycle progression in all organisms of the three domains of life: Bacteria, Archaea, and Eukaryota. This process begins by the assembly of a multiprotein complex at a predefined locus (multiple loci in Archaea and Eukaryota) on a chromosome, which is called the origin(s) of chromosomal replication (*ori*, in bacteria called *oriC*) [1,2]. The main roles of these nucleoprotein initiation complexes are to recognize the *ori* site, to distort the double helix, and to provide a platform for the assembly of the multiprotein replication machinery, termed the replisome, that will synthesize the nascent chromosome [3,4]. Chromosomal replication is highly regulated, mainly at the first step (initiation), to ensure that DNA replication does not begin under conditions that prevent the cell from completing the process, thus preventing the cell from dividing and producing a viable offspring cell [5,6].

The general mechanism of replication initiation is similar in all organisms. However, the number of initiation complexes per chromosome, initiation complex composition, protein-protein and protein-DNA interactions between initiation complex components, and check-point steps vary among organisms, with greater differences occurring among more unrelated taxonomic groups [3,4]. It is assumed that the molecular mechanism of replication initiation and its control are simplest in bacteria and most complex in Eukaryota. Indeed, the composition of the initiation complex in bacteria is less intricate than in organisms from the other two domains of life [1]. Nonetheless, the bacterial initiator protein DnaA is highly specialized, such that it can perform the functions of distinct subunits of Archaeal and Eukaryotic initiation complexes. For example, all initiators, including bacterial DnaA, Archaeal Orc1/Cdc6, or Eukaryotic Orc1-Orc6 origin recognition complex (ORC), recognize *ori* sites. However, in contrast to the last two, which are unable to melt DNA, only DnaA unwinds DNA and recruits other replisome proteins, especially the replicative helicase DnaB, to the newly formed single-stranded replication eye [7,8]. The DnaA protein and *oriC* are also the main factors controlling the assembly of the initiation complex or are subjected to control mechanisms that restrict the number of replications to one per cell cycle [6,9,10]. It is noteworthy that in some species, e.g., *Escherichia coli* or *Bacillus subtilis*, DnaA also serves as a transcription factor [11,12]. Thus, DnaA is a multifunctional protein, which is reflected by its complex structure and structure-function related activities.

## 2. Bacterial DnaA—General Overview of the Structure and Function

**Figure 1:**
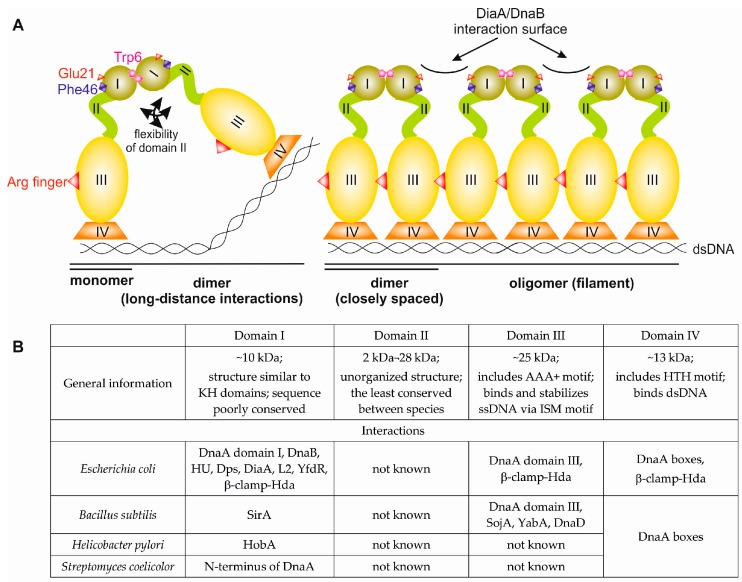
Domain structure of bacterial initiator protein DnaA. (**A**) A schematic overview of DnaA domains and their activities in orisome formation. Crucial residues involved in domain I dimerization (*E. coli* Trp6) and DnaB binding (*E. coli* Glu21 and Phe46) are marked. An arginine finger (*E. coli* Arg285), an ATPases Associated with diverse cellular Activities (AAA+) family-specific motif that recognizes ATP bound to an adjacent subunit in a multimeric complex, is also depicted. (**B**) General information about motifs, activities, and interacting partners of DnaA domains.

**Figure 2:**
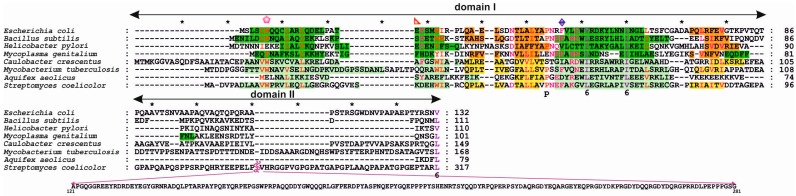
Sequence alignment of DnaA domains I–II from selected bacterial genera. The sequences were aligned using Profile ALIgNmEnt (PRALINE) [39]. Secondary elements of domains I–II are marked in green (α-helices) and brown (β-strands). Dark green and dark brown correspond to experimentally resolved structures of *H. pylori* (pdb 2WP0), *E. coli* (pdb 2E0G), *B. subtilis* (pdb 4TPS), and *M. genitalium* (pdb 2JMP) DnaAs; light green and light brown correspond to predicted secondary structures of *C. crescentus*, *M. tuberculosis*, and *A. aeolicus* DnaAs. The coloured fonts indicate conserved residues in domain I (violet, red, and pink from highest to lowest conservation, respectively); non-conserved residues are shown in black. Conserved residues involved in domain I dimerization (*E. coli* W6 (Trp6)), DnaB binding (*E. coli* E21 (Glu21), and F46 (Phe46)) are marked by a pink pentagon, red triangle, and violet peen, respectively; these symbols correspond to Figure 1.

To form a bacterial initiation complex, often called an orisome, DnaA binds to DNA at *oriC* and employs protein-protein interactions between protomers to assemble into a helical filament that is capable of opening double-stranded DNA (dsDNA) at the DNA unwinding element (DUE) [13]. DnaA is encoded by the *dnaA* gene, which is found in nearly all bacterial species. Exceptions include a few endosymbiotic bacteria, such as *Azolla filiculoides*, *Blochmannia floridanus*, and *Wigglesworthia glossinidia*, which lack a functional *dnaA* gene. In these bacteria, the initiator protein and mechanisms of initiation of chromosomal replication remain unidentified [14,15,16]. The DnaA proteins in bacteria characterized thus far vary in molecular weight between 47 kDa and 73 kDa (399-amino acid *Aquifex aeolicus* DnaA and 656-amino acid *Streptomyces coelicolor* DnaA, respectively). DnaA is composed of four structural and functional domains (Figure 1). The C-terminal domain IV encompasses approx. 120 amino acids (~13 kDa) and, together with domain III (approx. 230 amino acids, ~25 kDa), constitutes the most conserved part of DnaA with regard to structure and function. Domain II, which links domain III and domain I, is the most diverse domain between species with respect to sequence and length, varying between approx. 20 amino acids (~2 kDa) in *Helicobacter pylori* and approx. 250 amino acids (~28 kDa) in *S. coelicolor*. However, it should be noted that some DnaA proteins, such as the *A. aeolicus* initiator protein, appear to lack domain II (Figure 2) [17]. N-terminal domain I is composed of approx. 75–110 amino acids (~8–12 kD) (74 amino acids in *A. aeolicus* DnaA, 90 amino acids in *E. coli* DnaA, 108 amino acids in *Mycobacterium tuberculosis* DnaA), and in contrast to a well-conserved secondary structure, its sequence is poorly conserved among unrelated bacterial species.

Domain IV is responsible for DNA binding via a helix-turn-helix motif (Figure 1). The domain recognizes 9-mer, non-palindromic DNA sequences called DnaA boxes that are clustered at *oriC* (*E. coli* consensus sequence: 5′-TTATNCACA-3′). Domain III belongs to the ATPases Associated with diverse cellular Activities (AAA+) class of proteins; upon interaction with adenosine triphosphate (ATP), but not adenosine diphosphate (ADP), domain III changes conformation to enable the protein to properly oligomerize into a filament. The structure of such a filament bound to dsDNA and the means by which DnaA melts *oriC* is not fully understood. Nonetheless, the interaction between DnaA monomers within the filament introduce a conformational change in the bound DNA to melt its double-stranded structure at the DUE [18,19,20]. Subsequently, multiple domain III’s of the filament bind to and stabilize single-stranded DNA (ssDNA) via initiator-specific motifs (ISMs) [18,21,22,23,24]. *E. coli* DnaA domain III, together with domain I, recruits DnaB helicase to an open complex and helps position the helicase onto the ssDNA [25,26]; however, DnaA interactions with DnaB helicase and helicase loaders vary among species [27,28,29,30]. It should be noted that filamentation is mediated by domain III and controlled by other proteins that interact directly with this domain, such as a complex of the beta subunit of the DNA polymerase III (β-clamp) and the protein homologous to DnaA (Hda) (β-clamp-Hda- complex) in *E. coli* and possibly in *Caulobacter crescentus* or the sporulation initiation inhibitor protein Soj and the initiation-control protein YabA in *B. subtilis* [31,32,33,34,35]. Interestingly, as shown for *E. coli* DnaA, domains III and IV are sufficient in vitro for opening the *oriC* region; i.e., proteins that lack domains I and II unwind *oriC* in vitro in a manner similar to that of the full-length protein [36]. However, N-terminally truncated DnaA does not support DNA replication in vitro and is not viable in vivo, which indicates that the N-terminal part of *E. coli* DnaA is required to maintain its function in bacterial cells. Indeed, it has been shown that DnaA domain I, similar to domain III, mediates interactions between DnaA monomers and interacts with other proteins, including the helicase DnaB (see below).

Although the N-terminal domain is crucial for DnaA activity in vivo, its role in orisome formation is the least understood of the four domains. The reason for that is, in part, related to the lack of structure of full-length DnaA. The structure of the N-terminal portion of DnaA [37,38], which consists of a largely unstructured domain II and independently solved structures of domains III–IV [13,17,22], does not allow us to predict how the N-terminal domain is positioned within the orisome and how domain I is oriented with regard to the C-terminal domains III and IV. Due to the flexible domain II, DnaA domain I appears to be structurally detached from domains III–IV; however, it does affect DnaA activity in the orisome. Moreover, domain I is sensitive to regulation by cellular proteins (Figure 1B) that appear to coordinate DnaA activity with the bacterial growth phase or cell cycle, stress, or unknown stimuli. Domain I possibly controls the transition from the initiation phase to the elongation phase in *E. coli* through mutually exclusive interactions with regulatory proteins and DnaB. Altogether, the findings indicate that domain I is important for the activity of DnaA at the orisome.

## 3. N-Terminus of Bacterial DnaA

### 3.1. Structures of Bacterial DnaA Domains I and II

**Figure 3:**
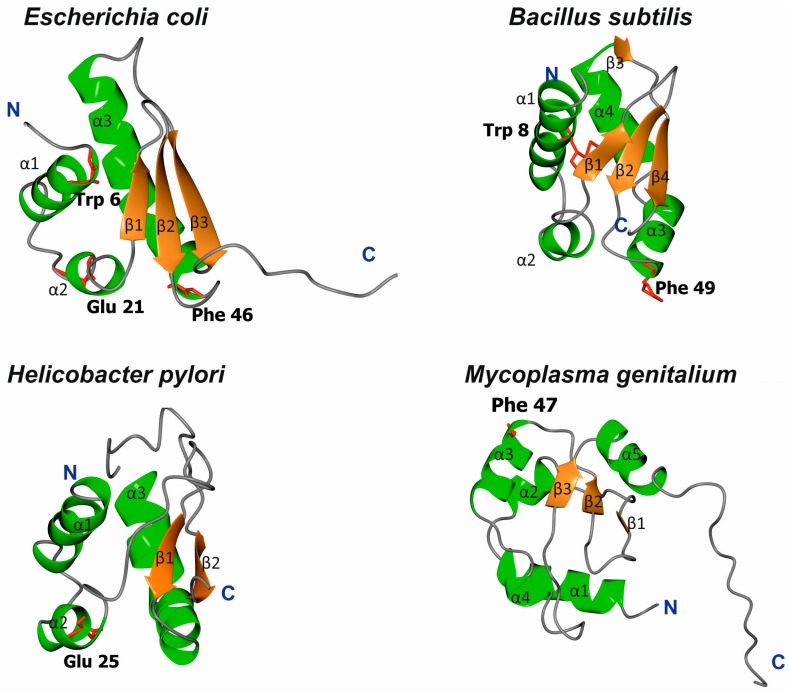
Ribbon diagrams of DnaA domain I in *E. coli* (pdb 2E0G), *B. subtilis* (pdb 4TPS), *H. pylori* (pdb 2WP0), and *M. genitalium* (pdb 2JMP). Residues involved in *E. coli* domain I dimerization (Trp) and DnaB binding (Glu, Phe) are marked (if conserved).

The structures of *E. coli*, *B. subtilis*, *H. pylori*, and *Mycoplasma genitalium* DnaA domain I have been solved; for the last, however, no functional analyses have been performed to date. Despite high sequence diversity (Figure 2), domain I is structurally conserved and consists of α-helices and β-strands (Figure 3). *E. coli* domain I is composed of 3 α-helices and 3 β-strands in the order of α1-α2-β1-β2-α3-β3 [37,38]. *H. pylori* DnaA is missing one β-strand between α1 and α2 [40], and *B. subtilis* DnaA contains an extra α4 helix between α3 and β3 [41]; *M. genitalium* contains two additional α-helices in the order of α1-α2-β1-β2-α3-α-β3-α [38] (Figure 3). Structurally, the α-helices and β-strands form distinct surfaces; an exception is for *M. genitalium*, in which the β-strands are packed between helices α1-α2 and α3-α4 at one site and α5 at the other. The β-strands comprise a β-sheet; however, the functional roles of the individual β-strands and entire β-sheet in domain I are unknown. The α helices are involved in different protein-protein interactions, and α1 of *E. coli* DnaA, together with a loop between β1-β2, forms a hydrophobic patch that engages in intermolecular interactions between the N-termini of DnaA monomers [37,42,43]. Nonetheless, this hydrophobic patch is not conserved among all DnaAs; for example, it is not present in *H. pylori* DnaA, and the N-terminus of this DnaA does not dimerize [40]. The α2 and α3 helices of *E. coli*, *H. pylori*, and *B. subtilis* DnaAs interact with other proteins (the DnaA initiator-associating factor DiaA and DnaB [37,44], the *Helicobacter* orisome binding protein A (HobA) [40], and the sporulation inhibitor of replication SirA [41], respectively), and despite a lack of sequence conservation, they are proposed to form structurally conserved protein-protein interaction surfaces utilized by regulatory proteins to control DnaA activity (see below) [41,44].

It has been reported that the structure of DnaA domain I is similar to the K homology domain (KH domain) [37,40]. KH domains interact with RNA and ssDNA nucleic acids, and affinity toward ssDNA or RNA is increased by the presence of multiple KH domains [45]. Additionally, the N-terminus of *E. coli* DnaA weakly interacts with ssDNA [37], though DnaA lacking domain I is able to unwind DNA and stabilize ssDNA via the ISM motif located in domain III [21,36,46]. Therefore, it remains unknown whether the KH motif plays any role in ssDNA binding upon unwinding of DNA by DnaA.

Domain II is unstructured and the most variable in sequence (Figure 2). Accordingly, there is little information about the possible motifs in regions that function in overall DnaA structure or function, especially within the context of mutual interdependence between domain I and domains III–IV.

### 3.2. Escherichia Coli DnaA Domain I

*E. coli* is a gram-negative, non-sporulating, facultatively anaerobic bacterium. Although *E. coli* constitutes a natural microflora in the lower intestine of warm-blooded organisms, including humans, some strains are pathogenic. This bacterium can survive and multiply outside of its host despite a decline in growth over time. The genomes of natural isolates of *E. coli* range from 4.5 to 6.0 Mb and encode approx. 4200–6500 genes. The bacterium has been used as a model organism for studying bacterial processes, including chromosomal replication and the cell cycle. Therefore, *E. coli* DnaA is one of the best characterized initiator proteins, especially within the context of structure-function relationships. In fact, studies on *E. coli* DnaA pioneered work on other initiators, including those in Archaea and Eukaryota. The resolved structure of *E. coli* DnaA domain I (1–86 aa) complements comprehensive biochemical data collected to date. It has been shown that domain I is engaged in numerous protein-protein interactions that include other DnaA monomers, as well as proteins that regulate DnaA activity at the orisome (DiaA, the histone-like protein HU, the ribosomal protein L2, the DNA-binding proteins from starved cells Dps, cryptic prophage protein YfdR, the β-clamp-Hda complex). Domain I of *E. coli* DnaA also participates in recruiting the replisome protein DnaB helicase; thus, it is important for the transition between the initiation and DNA synthesis (elongation) phases of replication.

**Figure 4:**
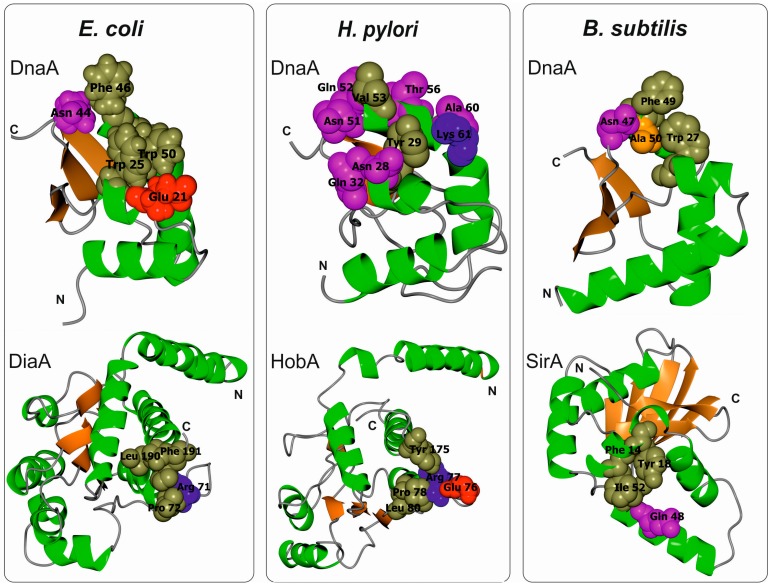
Ribbon diagrams of *E. coli*, *H. pylori*, and *B. subtilis* DnaA domain I and cognate interacting partners: DiaA (pdb 4U6N), HobA (pdb 2WP0), SirA (pdb 4PTS), respectively. Residues most important for complex formation are indicated by color-coded spheres (magenta—polar, orange—small non-polar, olive green—hydrophobic, red—negative charged, blue—positive charged).

The amino acids important for domain I head-to-head dimerization have been mapped to a patch formed by helix α1 and the loop between β1 and β2 (Figure 2 and Figure 3; amino acids leucine 5 (Leu5), tryptophan 6 (Trp6), glutamine 8 (Gln8), cysteine 9 (Cys9), Leu10, and Leu33) [37,42,43,47,48]. Regardless, how these interactions impact the structure and function of the entire DnaA protein, especially within the context of the assembled orisome, is still not fully understood. It has been suggested that N-terminal domains of *E. coli* DnaA, possibly due to dimerization of domain I, mediate long-distance interactions between DnaA monomers (Figure 1), similar to *S. coelicolor* (see below), and that this interaction facilitates or stabilizes DnaA binding to distantly located DnaA binding sites [49,50]. Dimerization might also be important to facilitate cooperativity of DnaA binding to closely spaced DnaA boxes, particularly for those with low affinity [49,51,52]. Indeed, domain I promotes DnaA oligomerization at *oriC*, possibly by bringing DnaA monomers into a closer contact so they can make a filament via domain III (Figure 1) [42,43]. The N-terminal domain is also required for DnaB loading [43]; DnaA defective in dimerisation via domain I (e.g., DnaA lacking the N-terminal domain or DnaA mutated at the amino acid Trp6, which is critical for domain I dimerization), is not able to load DnaB onto an open complex despite the fact that it can unwind DNA and bind to DnaB via a second interaction surface located at domain III [36,43,53]. It was suggested that dimerized domain I of DnaA oligomers at *oriC* provides an array of sites that, together with domain III, stably bind to DnaB and help load helicase onto ssDNA (Figure 1) [23,37]. Indeed, DnaB interacts with DnaA domain I via the amino acids glutamic acid 21 (Glu21) and phenylalanine 46 (Phe46), which are located on helix α2 and α3, respectively, i.e., at the region opposite from the α1 dimerization surface (Figure 3 and Figure 4) [36,37,53]. Such localization of surface interaction allows domain I to simultaneously dimerize and interact with DnaB.

As they are also engaged in interactions with DiaA and Hda regulatory proteins, DnaA helices α2 and α3 exposed to protein surfaces appear to be a hot spot for protein-protein interactions. DiaA is found in many bacterial species [54,55]. Although *E. coli* DiaA is not essential in vivo, it stimulates chromosomal replication, controls synchrony of initiation events, and ensures that the process is coordinated with the cell cycle [56]. Upon orisome formation, the DiaA tetramer simultaneously binds to multiple DnaA molecules and stimulates the assembly of DnaA onto *oriC*, which in turn facilitates the unwinding of the *oriC* duplex DNA [55]. In particular, amino acids Glu21 and Trp25 on α2 and asparagine 44 (Asn44), Phe46, and Trp50 on α3 are important for DiaA binding (Figure 4) [31,44,55]. Moreover, it has been shown that DiaA and DnaB compete for binding to DnaA and that DiaA bound to DnaA inhibits the DnaA-DnaB interaction and DnaB loading onto DnaA multimers at *oriC* [44]. These results demonstrate that DiaA controls DnaB loading [44,57]. The possible mechanism that regulates DiaA binding to DnaA is not known; however, it has been suggested that unknown cellular factors control DnaA-DiaA interactions [44].

Hda plays a pivotal role in regulating DnaA activity via a mechanism called RIDA (regulatory inactivation of DnaA). Hda consists of an N-terminal β-clamp-binding consensus sequence and the AAA+ domain, which shares homology with DnaA domain III. Hda-ADP in a complex with a β-clamp of DNA polymerase III interacts with DnaA domains I, III, and IV shortly after initiation [31,58], and inter-AAA+ interactions between domain III of *E. coli* DnaA and Hda stimulate the hydrolysis of ATP bound to DnaA [31,59]. DnaA-ADP is not able to properly oligomerize and unwind DNA; thus, it is inactive for initiation until it becomes reactivated into DnaA-ATP, which occurs either by DnaA de novo synthesis or by the interaction of DnaA-ADP with DnaA-reactivating sequences (DARS) or phospholipids (see below) [6,60,61]. Interactions between domains I and IV with Hda likely stabilize the complex and promote interactions between the AAA+ domains. In particular, DnaA mutated at Asn44 or lysine 54 (Lys54) located on helix α3 is insensitive to RIDA in vitro and in vivo [31]. Interestingly, *E. coli* domain I has also been proposed to participate in the transition of DnaA-ADP into DnaA-ATP, which is able to initiate replication [62]. Such an exchange of nucleotides, called rejuvenation, is promoted by the interaction between DnaA domain III and acidic phospholipids in the cell membrane [61]. However, it has recently been demonstrated that this process strongly depends on DnaA protein membrane occupancy, which affects the functional state of DnaA [62,63]. It was proposed that domain I is particularly important for rejuvenation associated with DnaA density-driven, cooperative oligomerization [62].

The molecular mechanisms of DnaA domain I interactions with HU, Dps, L2, and YfdR, and their roles in the initiation of chromosomal replication are much less understood than those described above. The HU protein is a DNA-binding protein that functions in compaction of the bacterial chromosome (by inducing DNA bends) and regulates DNA-related processes, including replication and transcription [64]. HU is composed of two subunits, α and β, that can form homo- and heterodimers. HU is known to stimulate in vitro DNA unwinding by DnaA, though the mechanism remains obscure [7,65]. Recently, it was shown that HU directly interacts with DnaA and that this interaction stabilizes DnaA oligomers assembled at *oriC* [66]. In particular, DnaA domain I preferentially binds to the α subunit of HU, either as an α2 or αβ dimer. In vitro, the α2 homodimer stimulates DNA replication more efficiently than αβ or β2. In vivo, the composition of the subunits in a dimer changes with the growth phase: the α2 dimer predominates during early log-phase growth but decreases to only approx. 5% of HU in the stationary phase [67]. Moreover, inactivation of the α but not the β subunit perturbs coordination between the initiation of DNA replication and the cell cycle. These findings suggest that HU facilitates initiation of chromosomal replication in *E. coli* during logarithmic growth.

In contrast to HU, proteins Dps, L2, and YfdR inhibit initiation [68,69,70]. Dps is synthesized upon exposure to environmental stress (e.g., oxidation, starvation) and protects DNA from oxidative stress via three intrinsic activities: DNA binding, iron sequestration, and ferroxidase enzymatic activity [71]. In vitro, Dps weakly inhibits DnaA-dependent replication of plasmids; however, the protein significantly (but not completely) inhibits chromosomal replication in vivo [68]. Interestingly, Dps synthesis is especially induced in oxygen-stressed cells during the logarithmic phase of growth. Under these conditions, Dps might be especially important for protecting replicating DNA and for inhibiting new rounds of DNA synthesis. However, it has been suggested that incomplete inhibition of replication initiation might allow for the synthesis of nascent DNA with mutations and, as a consequence, an increase in genetic variation within a population in response to oxidative stress [68].

L2 is a ribosomal protein that has recently been shown to interact with the N-terminus of DnaA [70]. In vitro, L2 and its truncated form, which lacks 59 N-terminal amino acids, destabilizes DnaA oligomers at *oriC* and thus inhibits DnaA-dependent DUE unwinding. Thus, L2 interferes with prepriming complex formation because it precludes DnaB loading, which is required for further replisome assembly. It has been suggested that L2 coordinates replication with transcription under specific, yet unknown, conditions.

YfdR, a protein encoded by a set of genes of the cryptic phage CPS-53, binds to domain I of *E. coli* DnaA in a Phe46-dependent manner [69]. Consistently, YfdR inhibits the binding of other Phe46-dependent proteins, DiaA and DnaB, to DnaA. YfdR also reduces the initiation of plasmid replication in vitro. Although the exact role of the YfdR protein is still not clarified, it has been suggested that the protein may regulate replication under specific stress conditions because the cryptic phage CPS-53 is involved in response to oxidative and acid stresses.

### 3.3. Bacillus Subtilis DnaA Domain I

*B. subtilis* is a gram-positive soil bacterium that sporulates under suboptimal growth conditions [72,73]. The genomes of natural isolates of *B. subtilis* range from 4.0 to 4.3 Mb and encode approx. 4000–4500 genes. Many *B. subtilis* cellular processes, including chromosomal replication, adjust to environmental conditions to promote vegetative growth, sporulation, or spore germination. Accordingly, a master Spo0A regulator, which is responsible for entry into sporulation, directly controls the activity of *oriC* [74,75] and indirectly regulates DnaA (see below). *B. subtilis oriC* is bipartite, i.e., it contains two clusters of DnaA boxes separated by a *dnaA* gene; both clusters are required for the initiation of chromosomal replication in vivo [76,77]. In vitro, DnaA binds to both sub-regions, acting as a bridge and looping out the *dnaA* gene [78]. *B. subtilis* DnaA-ATP has been shown to interact with *oriC* in a manner characteristic of AAA+ proteins; upon orisome assembly, DnaA-ATP forms a helix-like structure that unwinds DNA and binds to ssDNA [33,46]. Domain III of *B. subtilis* DnaA has a predominant role in DnaA filament assembly and is thus a target for binding numerous regulatory proteins, such as Soj, YabA, and the primosomal protein DnaD, none of which is found in *E. coli* [33,34,79]. In fact, *B. subtilis* DnaA domain III is the best characterized domain of the entire DnaA protein, whereas the roles of the other domains in the formation and activity of the initiation complex are much less understood. Knowledge of the role of the *B. subtilis* N-terminal domains (1–86 aa domain I, 87–111 aa domain II) in orisome assembly is particularly scarce. It is known that the N-terminal domains are not required for filament formation and ssDNA binding by *B. subtilis* DnaA in vitro [46], though it remains unclear whether *B. subtilis* DnaA domain I dimerizes. Most residues involved in the dimerization of *E. coli* DnaA domain I are conserved in *B. subtilis* DnaA (Figure 2 and Figure 3), and 22 amino acids of the N-terminus of the latter can functionally replace the 20 N-terminal residues of the former (i.e., helix α1) [48]. Such a hybrid protein complements the temperature-sensitive (Ts) growth phenotype of the dnaA46 mutant strain WM2063, though *E. coli* DnaA lacking 23 N-terminal amino acids is unable to complement this Ts strain. This suggests that the interaction between molecules of *B. subtilis* DnaA via domain I may occur and play a role in formation of the DnaA-*oriC* complex. This hypothesis is supported by the fact that SirA, which interacts with domain I of *B. subtilis* DnaA, displaces the initiator protein from *oriC* when incubated with the DnaA-*oriC* complex [80]. In vivo, SirA is produced under Spo0A∼P regulation and inhibits new rounds of replication prior to sporulation [80,81]. SirA forms a heterodimer with domain I of DnaA via interaction with initiator protein α2 and α3 helices. In addition, certain amino acids in domain I (Trp27, Asn47, Phe49, and alanine 50 (Ala50)) were shown to be especially important for interaction with SirA [41,82] (Figure 4). It is noteworthy that SirA also interacts with domain III [83] and, together with domain III-binding Soj and *oriC*-interacting Spo0A, controls *B. subtilis* chromosomal replication and coordinates replication during the transition from a vegetative to dormant state [74,83,84].

Unlike in *E. coli*, *B. subtilis* DnaA domain I appears to play no role in helicase recruitment into an open complex. Thus far, no interactions between *B. subtilis* DnaA domain I and helicase DnaC or helicase loading proteins (a loader—DnaI, a co-loader—DnaB, and an assisting protein—DnaD; please note the differences in helicase-related nomenclature; DnaD interacts with domain III of DnaA) have been reported [29,85]. Moreover, *B. subtilis* helicase is loaded onto ssDNA via a “ring-making” mechanism, which is different from the “ring-breaking” mechanism in *E. coli* [86,87]. Thus, distinct protein-protein interactions might be involved in helicase assembly into an open complex.

### 3.4. Helicobacter Pylori DnaA Domain I

*H. pylori* is a gram-negative pathogenic bacterium that resides in the human stomach, a relatively stable, albeit hostile, ecological niche [88,89]. The genomes of natural isolates of *H. pylori* range from 1.5 to 1.7 Mb and encode approx. 1400–1800 genes, with only a few regulatory proteins controlling cellular processes [90,91]. *H. pylori oriC* resembles *B. subtilis oriC*, i.e., it is bipartite and consists of two clusters of DnaA boxes, *oriC*1 and *oriC*2, separated by a *dnaA* gene [92]. The structure of *H. pylori oriC* and DnaA-DNA interactions have recently been well characterized [92,93,94,95], but there are limited biochemical data for *H. pylori* DnaA, particularly concerning domain III. For instance, it is not known whether *H. pylori* is regulated by ATP binding and hydrolysis, and no protein homologous to Hda has been found in *H. pylori*. Moreover, no proteins interacting with domain III of *H. pylori* DnaA have been identified thus far. As domain III is highly homologous among species, it likely forms a filament that is typical of DnaA. The N-terminus of *H. pylori* DnaA has been relatively well characterized. It comprises 110 amino acids (1–90 amino acids domain I, 91–110 amino acids domain II) and does not self-associate [40], possibly due to structural obstacles that may preclude dimerization. These obstacles include a shorter helix α1, a lack of conserved Trp6, and a positively charged (non-hydrophobic) area of interaction. *H. pylori* DnaA domain I interacts with HobA, a protein essential for *H. pylori* survival. To date, HobA is the only known protein that interacts with DnaA, and it influences DnaA assembly at *oriC* [96,97]. Indeed, HobA binding to DnaA stimulates DnaA oligomerization at *oriC*1 [54]. Despite low sequence homology, HobA is a structural and functional homologue of *E. coli* DiaA [54,98]. Similar to DiaA and SirA, HobA interacts with DnaA helices α2 and α3 [40], and residues tyrosine 29 (Tyr29), Asn28, and Gln32 on α2, and Lys61, valine 53 (Val53), Gln52, Asn51, Thr56, and Ala60 on α3 have been shown to be involved in interactions with HobA (Figure 4). However, DiaA and HobA cannot substitute for each other in vitro or in vivo because DiaA–*E. coli* DnaA and HobA–*H. pylori* DnaA interaction surfaces co-evolved [54]. Despite the high functional homology between DiaA and HobA, the dynamics of HobA/DiaA-stimulated oligomerization differ. HobA enhances and accelerates *H. pylori* DnaA binding to *oriC*, whereas DiaA increases but decelerates *E. coli* DnaA binding to *oriC*. Interestingly, the kinetics of responses involving domains III–IV do not depend on the stimulating protein (DiaA or HobA). In a hybrid system in which *E. coli* domain I was fused to domains II–IV of *H. pylori* DnaA (Ec^I^Hp^II-IV^DnaA), DiaA stimulated Ec^I^Hp^II-IV^DnaA in a manner similar to that of HobA stimulation of *H. pylori* DnaA, though with a sensitivity characteristic of DiaA [54]. This suggests that HobA or DiaA binding to cognate DnaA stimulates subsequent interaction, possibly between domain III, and that an induced response depends on domain III, the activity of which apparently differs slightly between these species.

It is not known whether the N-terminus of *H. pylori* DnaA or any domain of the DnaA protein participates in helicase loading onto an open complex because no DnaA-DnaB interactions, either between isolated proteins or within an orisome, have been shown thus far. Glu21, which is important for interactions of *E. coli* DnaA with *E. coli* DnaB, is present in *H. pylori* (Glu 25), but Phe46 is missing. It should be noted that *H. pylori* DnaB helicase is atypical, and unlike bacterial hexameric helicases, it forms a dodecamer that dissociates into hexamers upon interaction with DnaG primase [99,100]. Regardless, the mechanism for DnaB loading onto an open complex is still unknown.

### 3.5. Streptomyces Coelicolor DnaA Domain I

*S. coelicolor* is a gram-positive soil bacterium. It possesses a large, 9 Mb chromosome encoding approx. 8300 genes, which is almost twice as large as the *E. coli* or *B. subtilis* chromosome. *S. coelicolor* grows as substrate mycelia, which differentiate into an aerial mycelium and spores upon nutrient depletion. The key elements of the initiation of *S. coelicolor* chromosomal replication, DnaA and *oriC*, have been identified, and their interactions have been characterized [101,102,103,104,105,106]. *S. coelicolor* *oriC* contains two clusters of DnaA boxes separated by a short spacer DNA [103]; in total, there are 19 DnaA boxes spread over nearly 1000 bp. The DnaA-DNA complexes formed on both sides of the DNA spacer interact with each other to form a hairpin-like structure [106]. Although this resembles DnaA binding to bipartite origins in *B. subtilis* and *H. pylori*, the number of distinct nucleoprotein complexes is higher in *S. coelicolor* (up to 4 complexes per hairpin) than in the other two bacteria (1 complex per loop), as visualized by electron microscopy [78,92,106]. *S. coelicolor* DnaA is one of the largest known DnaA proteins (656 amino acids) due to the presence of a long domain II, which comprises an additional stretch (approx. 150 amino acids) of predominantly acidic amino acids. Such an exceptionally large domain II should enable DnaA dimers or oligomers to interact with distantly located DnaA boxes to establish a functional nucleoprotein complex. Domain I of the *S. coelicolor* DnaA protein dimerises [106], and together with domain III it participates in DnaA oligomerization [105,106]. It is possible that domain I mediates interactions between DnaA bound to distal DnaA boxes, whereas domain III mediates interactions between closely spaced boxes [106]. In addition, DnaA lacking domain I aggregates strongly upon DNA binding; thus, domain I should support the correct DnaA structure upon orisome formation [106]. Nonetheless, there is no detailed information concerning possible interaction surfaces or amino acids that participate in domain I intermolecular interactions, and there are no known proteins that interact with *S. coelicolor* DnaA. Thus, further studies are required to gain insight into protein-protein interactions that lead to assembly or regulation of a functional *S. coelicolor* orisome.

### 3.6. DnaA Domain II

Domain II was initially regarded as only a flexible linker that joins domain I with domains III–IV. However, it has been suggested that “nonessential” regions of domain II may be transiently involved in DnaB recruitment, and this domain, similar to DiaA, is presumably required to promote optimal helicase loading [107]. Moreover, domain II can be extended, and it tolerates the insertion of structured fragments. This was shown in *E. coli*, whereby green fluorescent protein (GFP) of 238 amino acids was inserted into domain II or into the C-terminal region of domain I (right after β3), without the loss of DnaA functionality in vivo [108,109]. In fact, it was the only location of GFP in DnaA that was tolerated by the *E. coli* protein. In addition, comprehensive deletion analysis within domain II of *E. coli* DnaA showed that at least 21–27 residues are required to sustain the correct conformation of the entire protein, possibly because they properly align domain I with domains III–IV [110]. Furthermore, deletions shortening *E. coli* domain II resulted in an under-initiation phenotype [107,111], which raises the question of how domain I and domains III–IV are aligned in proteins that have almost no existing domain II. Because domain I plays an important role in the cooperative binding of DnaA molecules at *oriC*, it is tempting to speculate that the length of domain II is adjusted according to the spacing between DnaA boxes. Regarding this hypothesis, the *S. coelicolor* DnaA protein can bind to widely spaced DnaA boxes due to the presence of a long domain II, whereas the *H. pylori* DnaA protein, with a relatively short domain II, binds to closely spaced *H. pylori* DnaA boxes [3]. It should reminded here, that the N-terminal domain I of *H. pylori* DnaA does not dimerise (Section 3.4, see also below), however, the direct interactions between the N-terminal domains of DnaA might be substituted by not-direct, HobA mediated, tetramerisation of DnaA [40,112].

## 4. Conclusions and Perspectives

The N-terminal domains of bacterial DnaAs are essential for full protein activity upon initiation of chromosomal replication, ensuring cooperativity of the protein in DNA binding and correct spatial assembly at *oriC*. This, in turn, is required for proper control of orisome activity with respect to further replisome assembly (e.g., DnaB loading) and the transition from the initiation to the DNA synthesis step. The N-terminal domains are also engaged in coordinating chromosomal replication with the cell cycle (e.g., sporulation) and other cellular processes (e.g., transcription) or environmental conditions (e.g., oxidative stress).

It should be noted that the N-terminal domains exhibit the least conserved sequence (Figure 2), and accordingly, it has been shown that the N-termini of DnaA from various species have different activities or interactions (Figure 1). The N-terminal domains likely evolved to meet the requirements of species that reflect differences in the structures of *oriCs*, the mechanisms of replisome assembly and the strategies of regulating DnaA activity. However, there are relatively few experimental data that assert the general features of the N-terminal domains with respect to the structure-function relationship of orisomes in different species. Nonetheless, dimerization and interaction with other proteins are the most conservative features of domain I. Domain II serves as a linker that coordinates the function of largely independent domains I, III, and IV.

It was experimentally shown that domain I in *E. coli* and *S. coelicolor* DnaAs dimerize. Helix α1 is crucial for dimerization in *E. coli*, but amino acids and interaction surfaces involved in *S. coelicolor* DnaA dimerization are unknown. In contrast, *H. pylori* DnaA domain I was shown not to interact, and there are no data regarding the dimerization of *B. subtilis* DnaA domain I. It was proposed that domain I dimerization and a sufficiently long, flexible domain II help to establish long-distance interactions. Thus, it was suggested that for some orisomes, domain I dimerization is not important when DnaA boxes are closely spaced at *oriC*, such as for *H. pylori oriC* [93,95,113]. However, *H. pylori* DnaA participates in long-distance interactions between DnaA-*oriC*1 and DnaA-*oriC*2 subcomplexes [92], raising the question of which domain (or domains) mediates the interactions between subcomplexes in *H. pylori*, *B. subtilis*, and other bipartite orisomes (e.g., mollicutes or Epsilonproteobacteria) [9,85,114].

Interaction of DnaA domain I with other proteins (*E. coli* DiaA, *H. pylori* HobA, and *B. subtilis* SirA) is mediated by helices α2 and α3, which likely comprise a common interface for protein-protein interactions (Figure 4). Interactions with DiaA and HobA are species specific, i.e., one protein cannot be substituted with another for interaction with DnaA in other species. Although it is not known whether SirA-DnaA interaction is also species specific, the amino acid sequence within the *B. subtilis* DnaA α2-α3 interface is quite different from that of *E. coli* and *H. pylori* DnaAs (Figure 4). In the structure-function relationship, it appears that proteins that bind multiple DnaA molecules, such as DiaA or HobA, stimulate DnaA oligomerization, whereas proteins that bind only a single DnaA protomer, such as SirA, destabilize DnaA oligomers. Multimerization of domain I might be important for cooperative binding of DnaA with DnaA boxes or for assembly of the multi-protomer interface for protein-protein interactions. When this interaction interface is released by DiaA/HobA, it can be further utilized by other proteins, such as when it is used by *E. coli* DnaB. However, proteins such as SirA might destabilize dimerization or the multi-protomer interface and thus preclude cooperative DNA binding or inhibit the loading of other proteins. It would be interesting to analyse how SirA affects oligomerization of hybrid DnaAs (*E. coli* (Bs^I^Ec^II-IV^DnaA) or *H. pylori* (Bs^I^Hp^II-IV^DnaA)), in which domain I is swapped for *B. subtilis* domain I. Such proteins should be able to interact with SirA, and this interaction could possibly destabilize orisomes formed by chimeric DnaAs.

Interaction between DnaA domain I and the helicase has only been demonstrated for *E. coli*. However, the interaction between DnaA domain III and helicase loader/loader assisting proteins appears to be more common in bacteria (DnaC binds to *A. aeolicus* DnaA [27], and DnaD interacts with *B. subtilis* DnaA [34,79]). It is reasonable to assume that by participating in helicase loading and activation, DnaA might be a key factor controlling the transition from initiation to elongation. More studies are required to reveal whether the binding between helicase and domain I of DnaA depends on the helicase loading mechanism (ring-making in *E. coli* vs. ring-breaking in *B. subtilis*), the loading proteins (*E. coli* DnaC, *B. subtilis* DnaI, or recently discovered DciA [30]), the *oriC* structure (*E. coli* mono- vs. *B. subtilis* bipartite), or other species-specific factors.

As mentioned above, domain I has various activities and has a different number and variety of interacting partners. The fact that there is a large discrepancy between the known activities exhibited by *E. coli* DnaA and initiators from other species is especially puzzling. Within this context, the N-terminus of *E. coli* DnaA appears to be an omnipotent domain. However, within the context of environmental challenges, physiology, and genetics, *E. coli* is not that different from other species, particularly *B. subtilis* or *S. coelicolor*. This makes it difficult to justify such an increase or decrease in the properties or interaction partners (seven, one, and zero DnaA interacting partners have been discovered thus far in *E. coli*, *B. subtilis*, and *S. coelicolor*, respectively—Figure 1). Nonetheless, these species have different life cycles. Thus, for example, because *E. coli* is unable to sporulate, it may require additional or different regulatory proteins to control chromosomal replication, whereas *B. subtilis* and *S. coelicolor* enter a dormant state under similar unfavourable conditions. Indeed, the initiation of *B. subtilis* chromosomal replication is controlled by Spo0A, SojA, and SirA, which are proteins associated with sporulation cycle control. Nonetheless, information is likely missing for many proteins that can interact with the N-terminal domain of DnaAs from other species, which, in turn, may regulate the initiation of chromosomal replication. For example, no interacting partners are known for *C. crescentus*, *S. coelicolor*, and *M. tuberculosis* DnaAs. It should be noted that in some bacteria, the number of proteins that regulate replication might be very low. For example, in *H. pylori*, a bacterium known for an overall limited number of regulatory proteins (compare approx. 30 proteins involved in signal transduction in *H. pylori* with approx. 300 and 1000 proteins in *E. coli/B. subtilis* and *S. coelicolor*, respectively [115]), the number of DnaA-interacting proteins might not be much higher than has been identified thus far. However, it is also possible that alternative pathways have been developed to control DnaA activity in *B. subtilis*, *S coelicolor, H. pylori*, and other bacteria. For example, it appears that *B. subtilis* DnaA is controlled primarily at domain III, whereas *C. crescentus* DnaA is primarily controlled at the levels of expression and proteolysis [116].

Functional and structural studies on *E. coli* DnaA-DiaA and *H. pylori* DnaA-HobA heterocomplexes have revealed relatively high specificity of interactions between initiation proteins [54]. This finding opens new possibilities for selective pathogen eradication by targeting essential protein-protein interactions involved in the initiation of chromosomal replication. Indeed, replication proteins are increasingly being considered as drug targets [117,118], among which species-specific domain I interactions appear promising. Thus, further studies will be important to increase our knowledge about the role of the N-terminus in controlling the initiation of bacterial chromosomal replication.
